# Effect of a Two-Step Temperature-Swing Synthesis on Coarse-Grained LiNiO_2_ Secondary Particles Characterized by Scanning Transmission Electron Microscopy

**DOI:** 10.1021/acs.chemmater.5c00108

**Published:** 2025-05-16

**Authors:** Thomas Demuth, Philipp Kurzhals, Shamail Ahmed, Felix Riewald, Michael Malaki, Johannes Haust, Andreas Beyer, Jürgen Janek, Kerstin Volz

**Affiliations:** † Marburg Center for Quantum Materials and Sustainable Technology (mar.quest) and Department of Physics, Philipps University Marburg, 35032 Marburg, Germany; ‡ Institue of Physical Chemistry and Center for Materials Research, Justus-Liebig-University (JLU), 35392 Giessen, Germany; § New Battery Materials and Systems, BASF SE, 67506 Ludwigshafen am Rhein, Germany

## Abstract

To enhance the range of electric vehicles, research is focused on increasing the nickel content in cathode active materials (CAM), which leads to higher practically achievable specific capacities. As a result, the material LiNiO_2_ (LNO) has attracted significant interest. In this study, a two-step temperature swing synthesis is employed to produce LNO secondary particles with large primary grains as CAM for solid-state batteries (SSBs). The synthesis involves sintering the material at 800 °C for 1 h, followed by an annealing step at a lower temperature for 6 h. Different batches of annealed LNO, using temperatures of 600 and 700 °C, are compared with unannealed LNO, utilizing various transmission electron microscopy (TEM) techniques. The annealing step contributes to smoother particle surfaces, reduced residual lithium species on particle surfaces, and increased lithium occupancy in the crystal lattice, resulting in higher discharge capacity during the initial cycles. However, higher annealing temperatures also lead to the formation of a thin rock-salt layer on the surface and internal misorientation, likely caused by thermal residual stress during cooling. These effects are more pronounced in the sample annealed at 700 °C compared to 600 °C. Despite the potential drawbacks associated with these factors, LNO annealed at 700 °C achieves the highest discharge capacity, indicating that the benefits of annealing outweigh its disadvantages, at least during the initial cycles.

## Graphical abstract

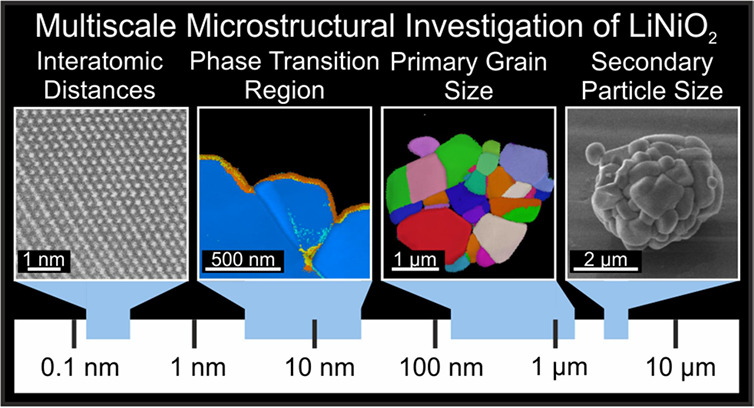

## 1 Introduction

The global push to reduce carbon emissions—particularly through the transition from vehicles powered by internal combustion engines to battery-electric vehicles, as well as the growing need to store energy from intermittent renewable sources—has led to an ever-increasing demand for high-capacity, high-energy storage solutions.^1,2^ While state-of-the art lithium-ion (Li-ion) batteries continue to improve, they are steadily approaching their energy-density limits.^3^ Consequently, new cell concepts are explored. Solid-state batteries (SSBs), which use solid electrolytes instead of the liquid electrolytes employed in conventional Li-ion batteries, could enable the use of lithium metal as negative electrode material. This could drastically increase energy density, making SSBs and their components a major focus of extensive research efforts.^4–7^ As the main contributor to the battery’s specific energy and cost, the positive electrode material—often addressed as cathode active material (CAM)—has been a key target for researchers.^8,9^ State-of-the-art CAMs are the layered transition metal (TM) oxides Li­(Ni_1–*x*–*y*_Co*_*x*_* Mn*_*y*_*)­O_2_ (NCM) and Li­(Ni_1–*x*–*y*_Co*_*x*_* Al*_*y*_*)­O_2_ (NCA) owing to their high specific capacity over 190 mAh g^–1^ as well as their relatively low costs.^10,11^ To further increase capacity and reduce the cost of CAMs, high nickel contents (≥95%) are being pursued.^12^ As a result, LiNiO_2_ (LNO) has attracted great research interest as limiting case.^13–15^ However, these Ni-rich CAMs suffer from reduced cycling stability due to microcracking along grain boundaries, irreversible phase transitions at the surface, and undesired side reactions with the electrolyte.^16–22^ To overcome these challenges, considerable efforts have been directed toward chemical stabilization approaches, such as doping with other elements and applying surface coatings.^23–26^ Other strategies include controlling the crystal orientation of the primary particles and utilizing materials with a TM concentration gradient.^27–30^ Furthermore, varying particle size and morphology have been found to influence the high first cycle irreversible capacity in LNO.^31,32^ In SSBs, smaller secondary CAM particles (≪10 μm) exhibit higher specific capacities in the first cycle compared to larger particles.^33^ Moreover, micron-sized single-crystalline particles are less prone to particle cracking and surface reactions as compared to secondary particles composed of primary particles with sizes of several hundred nm, which benefits cycle life.^34,35^ This is especially important in SSB applications, as particle cracking leads to voids between the CAM and the rigid solid electrolyte, resulting in the degradation of charge and discharge kinetics.^36,37^

In this study, we explore a synthesis route to prepare small secondary LNO particles (<5 μm) with large, approximately micron-sized monolithic primary grains. For material preparation, a two-step temperature-swing synthesis, as described in the publication by Qian et al., was employed.^38^ While high temperatures during sintering promote grain growth, they also induce cation mixing, which can negatively impact the electrochemical performance. To address this, a temperature-swing synthesis approach was employed. In this method, a high-temperature sintering step is applied to enlarge the grain size, followed by a lower-temperature annealing step for an extended period of time to complete the lithiation process and enhance structural ordering. In our study, the high-temperature sintering was performed at 800 °C for 1 h, while the lower temperature annealing was conducted at 600 and 700 °C for 6 h to investigate the influence of the annealing step and chosen temperature on structure, morphology, and electrochemical performance of the LNO particles. Samples annealed at 600 and 700 °C were thoroughly examined and compared to unannealed LNO, which did not undergo the additional annealing step. For clarity, these samples will be labeled as LNO-600, LNO-700, and u-LNO, respectively. The surface morphology and composition were analyzed using scanning electron microscopy (SEM) and titration of residual lithium compounds, while lithium occupancy in the crystal lattice was determined from powder X-ray diffraction (PXRD) measurements. Additionally, the materials were tested electrochemically to assess their discharge capacity in a coin cell setup using lithium metal as anode. The inner microstructure of the particles was investigated using various electron microscopy techniques that complement each other to provide comprehensive insights. High-angle annular darkfield (HAADF) scanning transmission electron microscopy (STEM) imaging was employed to capture high-resolution images of the LNO particles displaying the atomic arrangement. While this technique allows direct visualization of the atomic structure and identification of lattice defects, it is limited to local observations of small sample areas, providing only limited statistical data. Furthermore, while the layered LNO phase (*R*3̅*m*) and the degraded NiO-like rock-salt structure (*Fm*3̅*m*) are fundamentally different in structure, they can appear similar when viewed along certain orientations. For instance, in projections such as LNO [241] and NiO [110], the atomic arrangements overlap in a manner that makes distinguishing between the two phases challenging. In such cases, complementary electron energy loss spectroscopy (EELS) measurements offer insights into the phases present, as the oxygen bonding environment in the layered LNO phase differs from that in the NiO rock-salt phase, and the absence of a lithium peak in NiO can offer clear analytical information.^39^ However, like STEM, EELS measurements are localized and probe only a small portion of the sample. While EELS mapping of areas is possible, mapping large areas with sufficient resolution is impractical due to long acquisition times. Moreover, the effects of misorientations cannot directly be probed. Scanning precession electron diffraction (SPED), on the other hand, can bridge the gap between the nano- and microscale, providing information on the local crystallite phase and orientation with step sizes of a few nanometers over micrometer-sized areas. Cross-checking SPED results with EELS sampling helps to validate the accuracy of these measurements, as prior knowledge of the existing phases in the material is necessary for accurate phase and orientation mapping.

Using the wide variety of measurement techniques mentioned above enabled us to perform a multi modal analysis over various length scales up to the micrometer range, while still obtaining nanometer resolution. The sizes of various structural features discussed in this study, including interatomic distances, phase transition regions at the surface, primary grains, and secondary particles, are exemplarily presented in Figure 1 to enhance the understanding of their scale for a broader audience.

**Figure 1:**
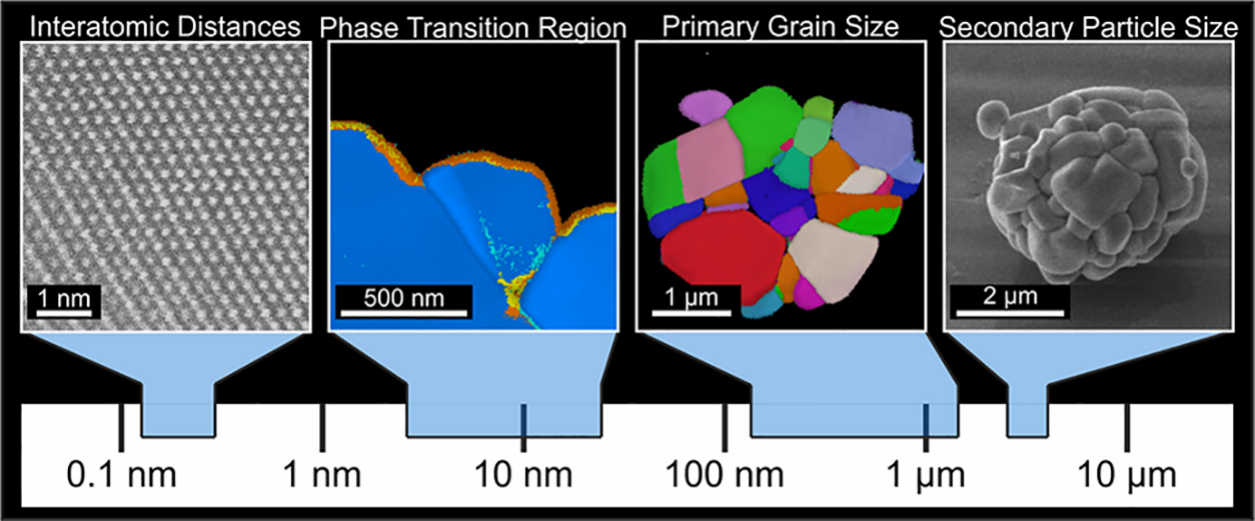
Size comparison of various structural features discussed in this study.

## 2 Materials and Methods

### 2.1 Synthesis of LNO Samples (Annealed and Unannealed)

The samples were calcined through a solid-state synthesis route starting from the base materials Ni­(OH)_2_ and LiOH·H_2_O. A commercial batch-type Ni­(OH)_2_ precursor (Hunan Zoomwe Zhengyuan Advanced Material Trade Co., Ltd.) with a secondary particle structure and *d*_50_ value of 4 μm was utilized. LiOH·H_2_O was used as a lithium source (Albemarle Corporation), which was ground before the synthesis with an air classifying mill to obtain particles of ∼10 to 20 μm. Thirty g of Ni­(OH)_2_ and the respective amount of LiOH·H_2_O to get the defined number of Li equivalents per mol of Ni of 1.02 were mixed using a laboratory blender (Kinematica AG). Afterward, this premix was filled into a ceramic crucible and fired in a laboratory box-type furnace (Linn High Therm GmbH). First, the temperature was ramped up to 400 °C and fixed for 4 h, and then the temperature was ramped up to the maximum calcination temperature *T*_max_ = 800 °C and was kept for 1 h. The u-LNO sample was cooled down right after the 800 °C step. In contrast, to prepare the two annealed samples LNO-600 and LNO-700, after the 800 °C step the temperature was lowered to 600 and 700 °C, respectively, and this temperature was kept for 6 h before cooling. For all steps, a heating rate of 3 °C min^–1^ was chosen. All experiments were run in a pure oxygen atmosphere (at a flow rate of 100 L per hour corresponding to about ten furnace-volume exchanges per hour). After the synthesis, the samples were left to cool to 120 °C and transported to a dry room (21 °C, dew point < −40 °C) inside a gastight box to mitigate reactions with ambient moisture and CO_2_, which lead to the formation of residual lithium compounds like LiOH and Li_2_CO_3_ on the surface of layered TM oxides.^40^ Handling of dry CAM powders was generally done in the dry room. While in a dry room exposure to CO_2_ cannot be excluded, the main catalyst required for Li_2_CO_3_ formation is moisture,^41^ which is mitigated here. Before the characterization of the materials, the powders were sieved using a sieve with a mesh size of 32 μm (Retsch GmbH) to deagglomerate the calcinate and sieve out impurities stemming from processing, like splinters of the ceramic crucibles or similar. Exclusively the sieved out fraction was used for experiments with sieving yields of more than 90%, resulting in batches of particles with homogeneous sizes.

### 2.2 Titration Analysis

Residual LiOH and Li_2_CO_3_ from LNO synthesis were determined by stirring a suspension of 2 g of material in 10 mL deionized water for 20 min under N_2_ atmosphere in a glovebox and titrating the resulting solution after filtration through a syringe filter with 0.1 M HCl solution (Bernd Kraft GmbH, Germany). To this end, a Titrando 808 automatic titrator (Deutsche Metrohm GmbH & Co. KG, Germany) and a glass pH electrode (Metrohm) were used. Two distinct equivalence points were observed during the titration process, which helped differentiate between LiOH and Li_2_CO_3_. The first protonation involved the hydroxide and carbonate ions, while the resulting bicarbonate was protonated during the second equivalence point.

### 2.3 Powder X-ray Diffraction (PXRD) Measurements

Powder X-ray diffraction (PXRD) measurements were performed using a Stoe Stadi P diffractometer with a molybdenum tube, a Johansson monochromator, and multi-MYTHEN detectors. Samples were measured in flame-sealed glass capillaries of 0.3 mm diameter and mounted on a sample holder and aligned both horizontally and vertically. This ensures that the sample material is positioned properly in the X-ray beam. A measuring range of 2−112° was taken and 8 individual measurements were carried out, which were summed after the measurement (measuring time of 30 min). Rietveld refinement of the crystal structure against powder diffraction data considering the instrumental contributions was performed using the DIFFRAC.TOPAS V6 software (Bruker AXS GmbH), and structural and microstructural (crystallite size and strain) information were obtained. The refinements were based on a rhombohedral α-NaFeO2 structure with the *R*3̅*m* space group in hexagonal lattice setting. The instrumental broadening was determined by measuring NIST 660c LaB6 in the same sample configuration. The model used for the fitting is based on Thompson-Cox-Hastings pseudo-Voigt convoluted with axial divergence asymmetry functions. Using the instrumental parameters, the sample contribution to the peak broadening was determined: with the angular dependence of the peak broadening related to a finite crystallite size described by the Scherrer equation, a volume-averaged value of the crystallite size was obtained. Refinement of the parameters of the structural model was done for consecutive iteration cycles until convergence was reached and the quality of the fit was checked by inspection of *R*_wp_ (weighted profile with all nonexcluded points), scale factor, sample displacement, a and c unite cell parameters, background (10th order Chebyshev coefficients), atomic displacement parameters, fractional atomic coordinate of oxygen *z*_Ox_, occupancy of Ni on Li site (assuming site remains fully occupied), *B*_iso_ (isotropic displacement parameter) of Li, Ni and of O. The “Li occupancy” was determined as the fraction of total Li ions being present on the Li sites in the crystal lattice, with a value closer to 100% indicating that the material comes close to the “ideal LiNiO_2_” structure without common structural defects such as Ni^2+^ ions on the Li sites. More information about the parameters and the Rietveld refinement is given in the Supporting Information as Supporting Information 1.

### 2.4 Electrochemical Cycling

Electrodes for electrochemical characterization were prepared by mixing the sample powders with conductive carbon (C65, Imerys Graphite & Carbon) and PVDF binder (Solef 5130, Solvay GmbH) at a 94:3:3 mass ratio. For this, a 7.5 wt % binder solution in *N*-methyl-2-pyrrolidone (NMP, BASF SE) was mixed with additional NMP and the conductive carbon and mixed for at least 24 min at 2000 rpm in a planetary mixer (ARE 250, Thinky Corporation). The CAM powders were added to the obtained slurry and were mixed for an additional 10 min. The solid content of the final slurries was 61 wt %. The slurries were cast onto an Al-foil (thickness 20 μm, Nippon Light Metal Co., Ltd.) using a box-type coater (wet-film thickness 100 μm, width 6 cm, Erichsen GmbH & Co. KG) and an automated coating Table (5 mm s^–1^, Coatmaster 510, Erichsen GmbH & Co. KG). The coated tapes were placed in a vacuum oven (VDL 23, Binder GmbH) and heated to 120 °C under a dynamic vacuum for drying overnight. The dried cathode tapes were compressed using a calender (CA5, Sumet Systems GmbH) at a set line force of 30 N mm^–1^ and a roller speed of 0.5 m min^–1^. Circular electrodes with a diameter of 14 mm were punched out using a high-precision hand-held punch (Nogamigiken Co., Ltd.). After weighing, the electrodes were transferred to an Ar-filled glovebox for cell assembly. An average loading of (8.0 ± 0.5) mg cm^–2^ and an electrode density of (3.0 ± 0.2) g cm^–3^ were obtained (corresponding to a porosity of ∼35%).

Coin cells were built using a 2032 geometry. The cell stack consisted of the cathode, a glass fiber separator (o̷ 17 mm, 300 μm thickness, GF/D, VWR International, LLC.) soaked with 95 μL electrolyte (LP57, BASF SE), and a prepunched lithium metal anode (o̷ 15.8 mm, thickness 0.58 mm, purity 99.9%, Shandong Gelon LIB Co., Ltd.). After assembly, the cells were crimped and closed in an automated crimper (Hohsen Corp.). The cells were then transferred to a climate chamber (Binder GmbH) and connected to a battery cycler (Series4000, MACCOR, Inc.). All tests were performed at 25 °C and the C-rate was chosen according to 1 C = 200 mA g^–1^. The cells were cycled between 3.0 and 4.3 V vs Li^+^/Li and the applied current was equal to C/20 in the first two cycles and C/3 in the following cycle.

Liquid electrolyte coin cells were chosen for testing due to the better reproducibility of liquid electrolyte cells over SSB lab cells, for which acquiring consistent reliable results remains difficult.^42^

### 2.5 Scanning Electron Microscopy (SEM)

Powder of the three types of LNO samples was dispersed on bare SEM stubs for observation in the SEM. The SEM images presented in this study were recorded with a HELIOS 5 Hydra CX. The beam current was 0.1 nA and the acceleration voltage was 5 kV. An Elstar through-the-lens detector (TLD-SE), located within the electron beam column, was used for image acquisitions utilizing secondary electrons (SE) in beam deceleration mode.

### 2.6 Sample Preparation by Focused Ion Beam (FIB)

TEM lamellae were prepared with a JEOL JIB 4601F dual beam system equipped with a Ga-ion beam. Initially, an approximately 200 nm thin tungsten layer was deposited with the electron beam, before a 2–3 μm thick tungsten layer was deposited with the ion beam using the gas injection system (GIS) of the FIB for protection. The particles were then attached to TEM copper lift-out grids utilizing a micromanipulator needle. In some cases, a dummy lamella made from silicon was used as a spacer between the grid and the particle. However, we did not find significant differences in sample quality between samples where the spacer lamella was used and not used. During thinning the ion beam energy was gradually reduced from 30 kV down to 5 kV until the lamellae had a thickness of less than about 100 nm.

### 2.7 Scanning Transmission Electron Microscopy (STEM) and Electron Energy Loss Spectroscopy (EELS)

STEM and EELS measurements were performed on a double Cs-corrected JEOL JEM 2200 FS (S)­TEM with an in-column omega filter operated at 200 kV acceleration voltage with a convergence angle of 15.07 mrad in STEM mode. The high-angle annular dark-field (HAADF) detector measures electrons in the angular range from 70 to 280 mrad. A TVIPS TemCam XF416FS camera was utilized for recording EELS spectra, which were evaluated and processed (correct zero-loss centering, background correction) using Gatan’s Digital Micrograph Software.

### 2.8 Scanning Precession Electron Diffraction (SPED)

SPED measurements were performed on a JEOL JEM-3010 TEM operated at 300 kV acceleration voltage. A NanoMEGAS P2000 ASTAR system was used to scan and precess the electron beam and a TVIPS TemCam XF416FS camera was used to acquire the four-dimensional data sets. The recorded video files were first converted into rectangular 4D data sets and these 4D data sets were subsequently converted into .bloc file format (compatible with ASTAR’s template matching software packages) by an in-house written Python code. These files were input into the software Index 2,^43^ which uses a pattern-matching algorithm for comparing recorded diffraction patterns with simulated patterns. The simulated patterns (.bnq files) were created from crystallographic files (.cif) of LiNiO_2,_ ^12^ Li_0.667_Ni_1.333_O_2,_ ^44^ Li­(NiO_2_)_2_ *Fd*3̅*m* (data retrieved from the Materials Project for Li­(NiO_2_)_2_(mp-25388) from database version v2025.02.12.post1.)^45^ and *Imma*,^44^ β-Ni_0.25_NiO_2,_ ^44^ γ-Ni_0.5_NiO_2_,^44^ δ-Ni_0.75_NiO_2_,^44^ Li_0.3_Ni_0.7_O,^46^ and NiO^47^ using the software DiffGen 2.^48^ The data was analyzed and processed into phases and orientation maps using the software MapViewer 2.^49^ Correlation Coefficient Maps (CCM) were generated from the 4D diffraction data using the Index software (maps in Figure 6, S4a,b,d–g and S5) as well as from in-house written python code (Figure S4c,h). Further analysis on the misorientation of the grains was performed using the software Esprit 2.3.^50^ For this purpose, the data was exported from MapViewer 2 as .ang files and imported into Esprit 2.3. The use of the TVIPS TemCam, instead of the standard Stingray camera commonly employed for acquiring SPED data by recording the fluorescent screen, results in significantly enhanced contrast in the diffraction patterns. This improved contrast enables the detection of even weak diffraction spots, leading to a substantial increase in pattern-matching accuracy.

## 3 Results and Discussion

Figure 2 depicts secondary electron (SE) images of two LNO secondary particles from each of the three sample types. The u-LNO particles exhibit a rough and furrowed surface, particularly at the edges of the primary particles, as highlighted in Figure 2a,b. Additionally, oval-shaped stepped terraces are prominent on some of the primary particles. The annealing process significantly alters the morphology of the particles. In LNO-600, the rough and furrowed structures at the edges are largely diminished. However, some particles still exhibit a rugged surface (Figure 2c) with larger and much broader features than those observed in u-LNO. Other particles, such as the one shown in Figure 2d, have a smoother surface but still exhibit large, oval-shaped stepped terraces. In contrast, the LNO-700 particles are predominantly smooth, with only a few remnants of the terraces visible (Figure 2e,f).

**Figure 2:**
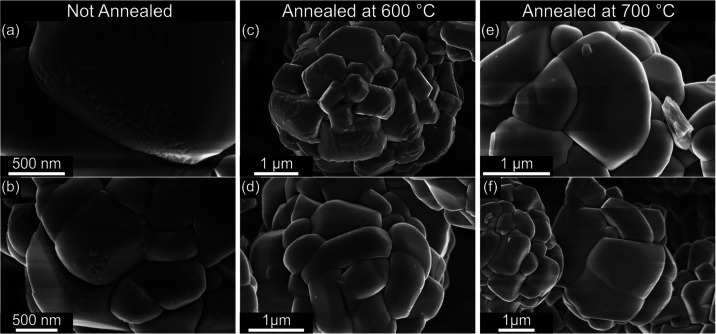
SEM SE images of two different LNO secondary particles for each sample type: u-LNO: (a, b); LNO-600: (c, d); LNO-700: (e, f).

Thus, the annealing process smooths the surface of the primary particles. The impact of primary particle surface roughness on the battery performance is uncertain. We presume that it may differ for different types of electrolytes used. For liquid and soft solid electrolytes, a rougher surface with increased surface area may enhance performance by improving interfacial contact and facilitating ion transport. Conversely, rigid solid electrolytes tend to form more effective contact with smoother surfaces, and furrows in this case may lead to void formation, compromising the integrity and performance of the solid–solid interface.

In addition to the morphological changes, the annealing step reduces the amount of reactive lithium species on the particles’ surfaces, as indicated by the titration results presented in Figure 3a. The amount of titratable LiOH on the LNO particle surface decreases by more than half from 0.92 wt % in u-LNO to 0.45 wt % after annealing at 700 °C. Similarly, the amount of titratable Li_2_CO_3_ is reduced from 0.59 to 0.43 wt %.

**Figure 3:**
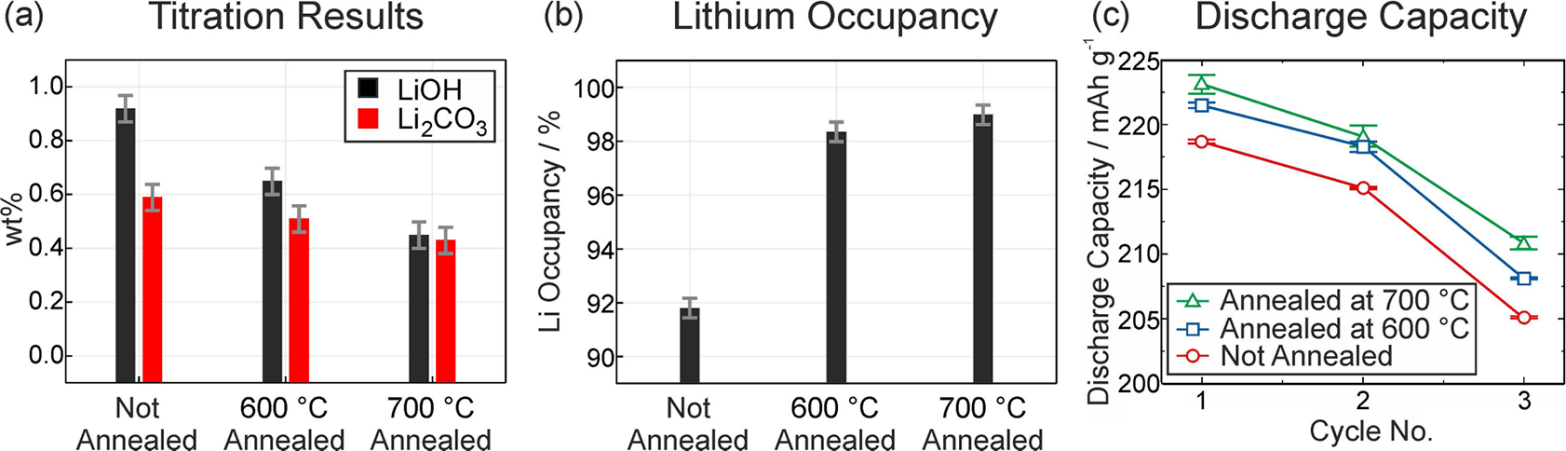
(a) Titration results showing the wt % of LiOH and Li_2_CO_3_ compounds on the particle surface of the different types of LNO. (b) Li occupancy of the different types of LNO as determined by PXRD. (c) Discharge capacity in the first three cycles of the three differently treated LNO samples. The error bars represent the variation between different cell tests.

While the amount of reactive lithium on the surface decreases during annealing, PXRD measurements (provided in Figure S1) reveal that lithium occupancy within the LNO structure, as shown in Figure 3b, simultaneously increases. For u-LNO, the lithium occupancy amounts to 92%. This finding is consistent with observations from literature, as achieving stoichiometric LiNiO_2_ during synthesis is challenging, often resulting in a “cation off-stoichiometric” LNO of the form Li_1–*x*_Ni_1+*x*_O_2_, where Ni^2+^ ions occupy lithium sites.^51–53^ In contrast to u-LNO, the Li occupancy increases to 98 and 99% for LNO-600 and LNO-700, respectively.^44^ Thus, we deduce that annealing at 600 and 700 °C reduces the value for *x*, as reactive lithium present on the particle’s surface is reinserted into the lattice, leading to a higher lithium occupancy in the bulk of the particle and a reduction in the amount of Ni^2+^ ions in the Li layer.

The lithium occupancy results align with our findings during electrochemical cycling. Coin cells were cycled between 3.0 and 4.3 V vs Li^+^/Li. The applied current was equal to C/20 in the first two cycles and C/3 in the following cycle. Figure 3c depicts the discharge capacities of the three types of LNO samples. u-LNO exhibits a first cycle discharge capacity of around 219 mAh g^–1^, while LNO-600 and LNO-700 show slightly higher discharge capacities of 222 and 223 mAh g^–1^, respectively. This trend also holds for the change of C rate to C/3 in the third cycle (u-LNO with 205 mAh g^–1^, LNO-600 with 208 mAh g^–1^ and LNO-700 with 211 mAh g^–1^). This supports our conclusion that reactive lithium on the surface moves back into the LNO lattice during the annealing process, leading to a slightly higher discharge capacity.

To correlate the observed morphological changes on the particle surface with their internal structure near the surface, SPED data sets were recorded for the three types of LNO. During SPED data acquisition, the electron beam is scanned over the sample and precessed around the optic axis at each scan point. The precession of the beam suppresses dynamical scattering effects, resulting in diffraction patterns that behave kinematical-like.^54^ These diffraction patterns are compared with simulated diffraction patterns of the expected phases using a template-matching algorithm, providing insight into the crystal phase and orientation of the investigated samples.^55^ For phase identification, we selected reference structures that have been experimentally observed or are expected to appear during the transition from the layered to the rock-salt phase. These include layered LiNiO_2_ (*R*3̅*m*), Li_0.667_Ni_1.333_O_2_ (*C*2/*c*), spinel Li­(NiO_2_)_2_ (*Fd*3̅*m* and *Imma*), β-Ni_0.25_NiO_2_ (*R*3̅*m*), γ-Ni_0.5_NiO_2_ (*Cmmm*), δ-Ni_0.75_NiO_2_ (*Fm*3̅*m*), and Li_1–*x*_Ni_1+x_O_2_ rock-salt phase (*Fm*3̅*m*).^44,56,57^ Additionally, for a more detailed investigation of the rock-salt phase, both pure NiO and a lithiated rock-salt phase (Li_0.3_Ni_0.7_O) were included.^46,47^ It is important to note that the matched phases do not represent definite identifications, but rather the closest structural match among the selected input phases within a continuous spectrum of structures ranging from layered LNO to fully delithiated rock salt NiO. Consequently, regions matched with NiO might still contain some lithium but are structurally closer to NiO than to Li_0.3_Ni_0.7_O. Phase maps of the surface of u-LNO, LNO-600, as well as LNO-700 are depicted in Figure 4a–c. Since the spinel phase Li­(NiO_2_)_2_ (*Imma*) was not identified in the pattern matching process, it is not included in the phase map labels. u-LNO grains consist almost exclusively of the layered LiNiO_2_ phase, with only small amounts of Li_0.3_Ni_0.7_O rock-salt phase present at the very surface. At the grain boundary, small amounts of Li_0.667_Ni_1.333_O_2_ and, β-Ni_0.25_NiO_2_ are detected. The annealed samples exhibit a multiple nanometers wide phase transition region at the surface. In LNO-600, the phase transition region of the large grain predominantly exhibits spinel Li­(NiO_2_)_2_ *Fd*3̅*m*, along with β-Ni_0.25_NiO_2_ and Li_0.3_Ni_0.7_O. Other grains mainly exhibit a Li_0.3_Ni_0.7_O surface layer. Again, we emphasize that the assigned phases are not definitive, but rather the closest approximation to the recorded patterns. This is evident in Figure S2. Here, the indexing software matches the β-Ni_0.25_NiO_2_ phase, even though it does not perfectly fit the experimental pattern. In contrast, layered LiNiO_2_ in the bulk of the grain matches the recorded pattern almost exactly. Thus, close to the surface, an intermediate phase is present that most likely still contains some lithium but is structurally closest to β-Ni_0.25_NiO_2_. The surface of LNO-700 does not exhibit spinel Li­(NiO_2_)_2_ and β-Ni_0.25_NiO_2_. Instead, mainly Li_0.3_Ni_0.7_O, along with an increased NiO content is identified, indicating a progressive formation of the rock-salt layer with advanced lithium loss for increasing annealing temperature. All other phases are only detected in negligible amounts. This observation aligns with previous reports in literature that higher temperatures during the material’s synthesis induce the formation of a NiO-like rock-salt layer on the surface of layered TM oxides.^12,18,58,59^

**Figure 4:**
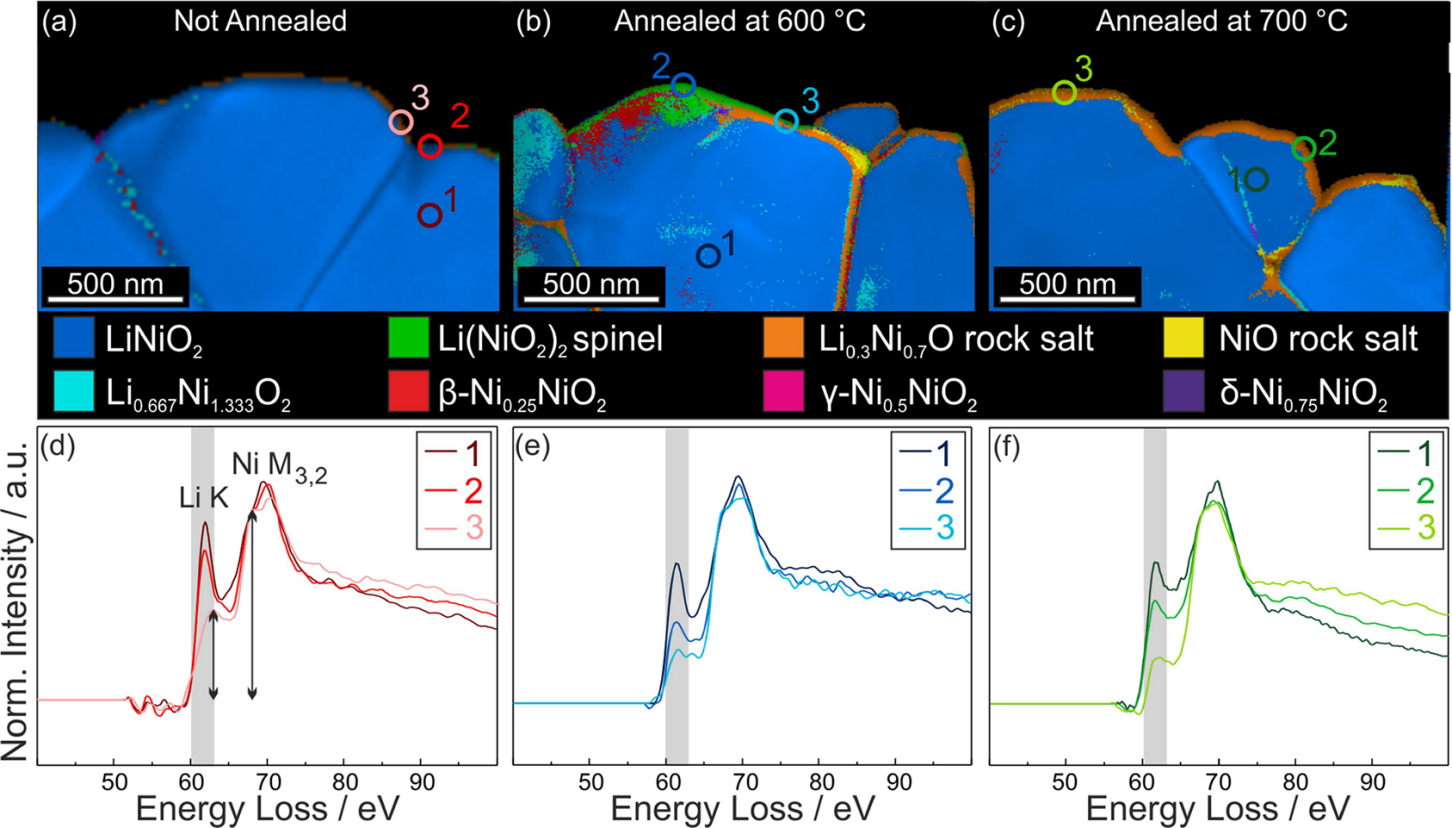
SPED phase maps overlaid on the index map of the surface of (a) u-LNO, (b) LNO-600, and (c) LNO-700. (d–f) EEL spectra of the Li K-edge (gray background) normalized to the Ni M_3_-edge at 67 eV recorded at the positions marked in (a–c).

Since the SPED results do not show definitive phases, we recorded EELS spectra at relevant positions on the same samples to obtain more detailed information on the sample. To reduce the electron dose during EELS acquisition we recorded spectra from square-shaped areas with a size of a few square nanometers. The spectra were normalized to the intensity of the Ni M_3_-edge at 67 eV to eliminate thickness effects and allow for better comparison between the spectra. As evident from the spectra shown in Figure 4d–f, the bulk regions of the particles, which were identified as the layered LNO phase by the SPED template matching algorithm, exhibit a distinct lithium K-edge peak at 61 eV (highlighted with a gray background) (spectra (d) 1, (e) 1, and (f) 1). In the bulk, we obtain Li K-edge to Ni M_3_-edge ratios >0.7. Interestingly, the Li K-edge/Ni M-edge ratio in the u-LNO bulk is approximately 0.9. This is significantly higher than the values reported by Park et al., who measured Li-K/Ni-M_3_ ratios >0.6 for the layered phase.^60^ We assume that the variation in Li K-edge to Ni M_3_-edge ratios in the bulk for different samples are influenced by the quality of the TEM specimen. Therefore, a quantitative comparison between spectra is only meaningful when performed within the same sample, not across different samples. The Li K-edge intensity of spectra recorded from regions closer to the surface qualitatively corresponds to the phases identified in the SPED maps. In u-LNO, region 2—matched with layered LNO—exhibits a Li K-edge/Ni M_3_-edge ratio of approximately 0.8, while region 3—matched with Li_0.3_Ni_0.7_O—shows a ratio of around 0.5. This results in a Li K-edge intensity ratio (region-to-bulk) of roughly 0.9 and 0.5, respectively. In LNO-600, region 2—matched with spinel Li­(NiO_2_)_2_ (*Fd*3̅*m*)—exhibits a Li K-edge/Ni M_3_-edge ratio of approximately 0.4, while region 3—matched with a mixture of spinel Li­(NiO_2_)_2_ and Li_0.3_Ni_0.7_O—shows a ratio of about 0.3. This corresponds to surface-to-bulk Li K-edge ratios of 0.6 and 0.4, respectively. For LNO-700, region 2—recorded from a surface region with a thin Li_0.3_Ni_0.7_O layer—exhibits a Li K-edge/Ni M_3_-edge ratio of approximately 0.6. Region 1—matched with a mixture of Li_0.3_Ni_0.7_O and NiO—shows a ratio of around 0.2. This results in surface-to-bulk Li K-edge ratios of 0.7 and 0.3 for regions 2 and 3, respectively. The measured intensities used to calculate the ratios are presented in Table S1 in the Supporting Information. As the SPED and EELS data were recorded using different microscopes, the assigned regions in Figure 4 may slightly deviate from their actual positions. HAADF STEM images showing the precise locations of the EELS scan areas are provided in the Supporting Information (Figure S3). Furthermore, since EELS data were collected in a dose-efficient manner over small areas rather than single points, contributions from more than one phase to the lithium peak cannot be excluded. Therefore, a direct quantification of SPED phases by EELS is only possible to a limited extent. Nevertheless, the EELS results show the same trend as the SPED data, supporting the conclusion that a phase transition toward a rock-salt phase occurs at the surface during annealing. With increasing annealing temperature, this transformation progresses further, accompanied by a gradual loss of lithium from the lattice. However, the measurements also indicate that the rock-salt layer remains partially lithiated, which may still allow lithium insertion and extraction during (dis)­charging.

Beyond the obvious differences in Li K-edge intensity, the shape of the broad peak at ∼69 eV differs between the layered and rock-salt structures. The difference in intensity between the Ni M_3_-edge at 67 eV and the peak at 69 eV decreases when the Li K-edge intensity is low and increases when the Li K-edge intensity is higher, as illustrated by the dashed lines in Figure 4d,e. EELS data recorded by Park et al. also demonstrate this trend.^60^ However, because lithium exhibits a postedge at 69 eV, it remains unclear whether the differences in peak shapes are due to an overlap of the Li postedge and the Ni M_2_-edge or a varying fine structure of the Ni M-edge peak for the different phases.^61^

To further analyze the rock-salt surface layer, we recorded high-resolution (HR-) HAADF STEM images. Figure 5a shows an approximately 10−15 nm wide rock-salt layer on the surface of the particle from LNO-700. Generally, the formation of a rock-salt layer in layered TM oxides is associated with particle degradation and subsequent capacity loss.^62–64^ However, recent studies suggest that the presence of disordered phases, Ni/Li antisite defects near the surface, and the formation of a sufficiently lithiated rock-salt phase prior to cycling can stabilize the surface and suppress further rock-salt formation during cycling, leading to enhanced electrochemical performance and improved cycling stability by passivating the surface.^65–71^ Our findings are more in line with the latter studies, as we observe a higher discharge capacity for the annealed sample with a more pronounced, but still lithiated, rock-salt structure compared to the unannealed sample (see Figure 3c). The increased presence of cation-mixed phases and rock-salt phase at the surface of LNO-700 does not appear to negatively impact its discharge capacity in the first cycles. As a rock-salt layer forms in situ, when LiNiO_2_ is delithiated over the H2–H3 phase transition plateau during cycling,^72^ the initial presence of a rock-salt layer before cycling might not be detrimental to the performance after all.^62–68^ Furthermore, as rock-salt phase formation is associated with oxygen evolution,^73^ it may be advantageous to control the rock salt formation during material synthesis rather than allowing it to occur in situ within the cell.

**Figure 5:**
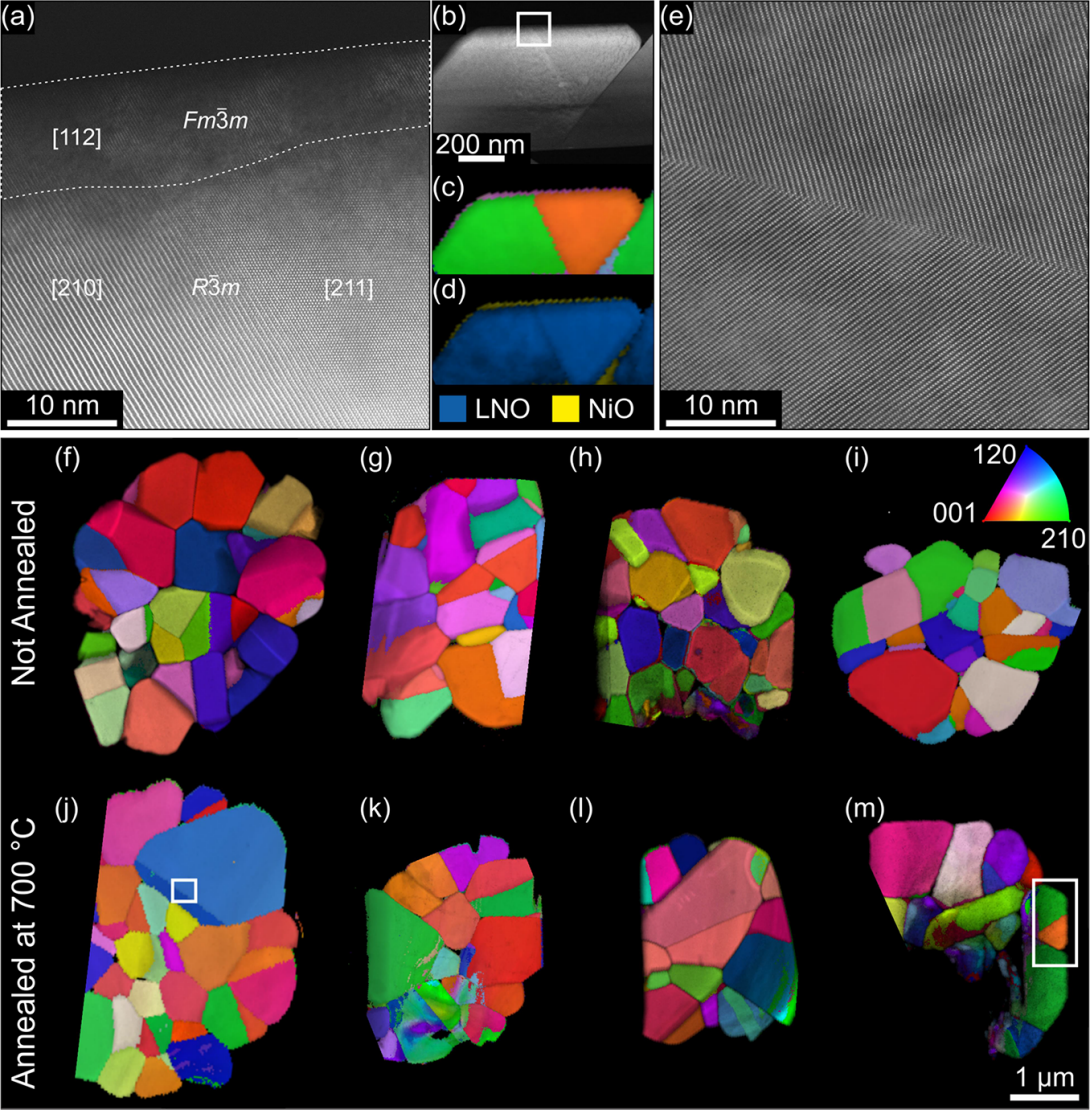
(a) HAADF HR-STEM image of the region marked in (b). (b) HAADF-STEM image of a primary-like particle within the LNO-700 particle depicted in (m). SPED (c) orientation and (d) phase map overlaid on the index map of the primary-like particle shown in (b). (e) HAADF HR-STEM image of the twin boundary marked in (j). SPED orientation maps (in z orientation) overlaid on the index map of (f–i) u-LNO and (j–m) LNO-700 particles. Areas of drastically varying orientations in particles (h, k, m) are artifacts from sample preparation by ion beam milling.

The observation of a rock-salt layer, which contains significantly less lithium than the LiNiO_2_ phase, at elevated annealing temperatures through SPED and EELS measurements appears to contradict the lithium occupancy results obtained from PXRD. While u-LNO shows the least amount of rock-salt phase, it has the lowest Li occupancy value. In contrast, LNO-700 exhibits the most amount of rock-salt phase at the surface but simultaneously the highest amount of Li occupancy.^46–48^ However, a rough estimation of the ratio between the layered bulk region and the rock-salt layer on the surface reveals that bulk Li occupancy changes outweigh Li loss on the surface. In a spherical secondary particle with a diameter of approximately 4 μm, a rock-salt layer of around 10 nm thickness at the particle surface constitutes for only about 1% of the total volume. This minimal proportion explains the negligible impact of rock-salt layer formation on Li occupancy during annealing at elevated temperatures.

In addition to the thin rock-salt layer on the LNO-700 particle’s surface observed in the HAADF-STEM image, two other crystallographic domains that merge seamlessly into each other are visible in Figure 5a. To identify the domains’ orientations and phases, which cannot unambiguously be deduced from the HAADF STEM image in Figure 5a alone, we recorded an SPED data set of the entire grain. The grain is depicted in an HAADF STEM image in Figure 5b. The orientation and phase maps obtained from the SPED data, presented in Figure 5c,d, respectively, identified the domains as the LiNiO_2_ layered phase in [210] and [211] orientation, which fits the HAADF STEM observation in Figure 5a. Thus, what appears to be a primary particle based on its shape and morphology at the micrometer scale (see Figure 5b) is a polycrystalline primary-like particle, as described by Lee et al., consisting of smaller coherently aligned grains.^74^ These particles contain coherent grain boundaries, including twin boundaries (TBs). These are reported to negatively affect the CAM’s performance by leading to crack and void formation, as well as phase transitions and subsequent performance decay during electrochemical cycling.^74–78^ Moreover, Nguyen et al. discovered that planar defects hinder Li reinsertion into the CAM, contributing to first cycle irreversible capacity.^79^ Given the detrimental effects of planar defects on CAM performance, we investigated, whether the annealing step influences their occurrence. While HR-STEM effectively images planar defects, as Figure 5a as well as the twin boundary found in an LNO-700 particle shown in Figure 5e illustrate, it provides limited statistical data. Therefore, we used SPED measurements, which allow for the examination of entire secondary particles and facilitate the detection of planar defects through orientation maps. For better statistical analysis, we investigated four particles each of u-LNO and LNO-700, as displayed in Figure 5f–m. It is evident that the occurrence of planar defects varies drastically among particles in both u-LNO and LNO-700 samples. Some u-LNO particles exhibit more planar defects than certain LNO-700 particles (e.g., the particle shown in Figure 5g compared to Figure 5k), and vice versa (e.g., the particle shown in Figure 5j compared to Figure 5h). Since no consistent trend emerges (i.e., one type consistently showing more planar defects than the other), it appears that the additional annealing step neither reduces nor promotes the formation of planar defects. We conclude that the additional annealing step does not significantly affect the presence of crystallographic defects within the particles, as the annealing temperature of 700 °C is likely insufficient to realign entire crystal domains within the twinned crystallite structure.

In addition to orientation maps, we generated correlation coefficient maps (CCMs) of each of the eight particles presented in Figure 5f–m from the SPED data, which are shown in Figure S4.^80^ While orientation maps reveal the orientation of entire crystal domains and are primarily sensitive to large orientation shifts, CCMs capture variations between adjacent diffraction patterns. In these maps, darker contrasts indicate a greater degree of misorientation between neighboring diffraction patterns, making CCMs a useful representation to easily detect planar defects and more subtle changes within regions of the same crystal orientation. While the orientation maps do not show significant differences between u-LNO and LNO-700 regarding the occurrence of planar defects despite the investigation of eight secondary particles in total, the CCMs of the different LNO types exhibit striking differences. The primary grains in u-LNO show no significant contrast changes, as the CCM of a u-LNO particle depicted in Figure 6a shows. In contrast, most grains in LNO-700 display dark lines, as seen in the CCM in Figure 6d. These grains exhibit a significantly higher degree of misorientation perpendicular to the lines compared to parallel to the lines, as the graph in Figure 6e reveals. In u-LNO, there is minimal misorientation within the grain regardless of direction (see Figure 6b). To represent the misorientation angle within the grains for a statistically relevant number of grains, we applied a color code to each individual grain of the respective LNO particles. Dark blue colors represent no misorientation relative to the position of the red cross in the center of the particle, while lighter blue to green indicates a higher degree of misorientation. We restricted the selection to true primary grains, excluding primary-like particles with significant misorientation across planar defects. The color-coded maps of both u-LNO (Figure 6c), and LNO-700 (Figure 6f) clearly show that grains in LNO-700 exhibit a higher degree of continuous low-angle misorientations compared to u-LNO, as evidenced by the greater presence of light blue and green hues. These continuous low-angle misorientations hint at lattice plane bending within a primary grain. Layered TM oxides are known to exhibit a degree of flexibility, allowing the planes to bend in response to internal stress without the formation of significant crystalline defects.^74^ Therefore, we conclude that the low-angle misorientations observed in LNO-700 in this case do not result in the formation of tilt boundaries and geometrically necessary dislocations, as reported by Xu et al. for LiCoO_2_ particles.^81^ To ensure that these features are not artifacts of sample preparation by FIB, we investigated four samples of each type. The CCMs of all eight LNO particles, provided in Figure S4, follow the same trend. Interestingly, low-angle misorientation lines are considerably less distinctive in the CCM for LNO-600 compared to LNO-700, as illustrated in Figure S5.

**Figure 6:**
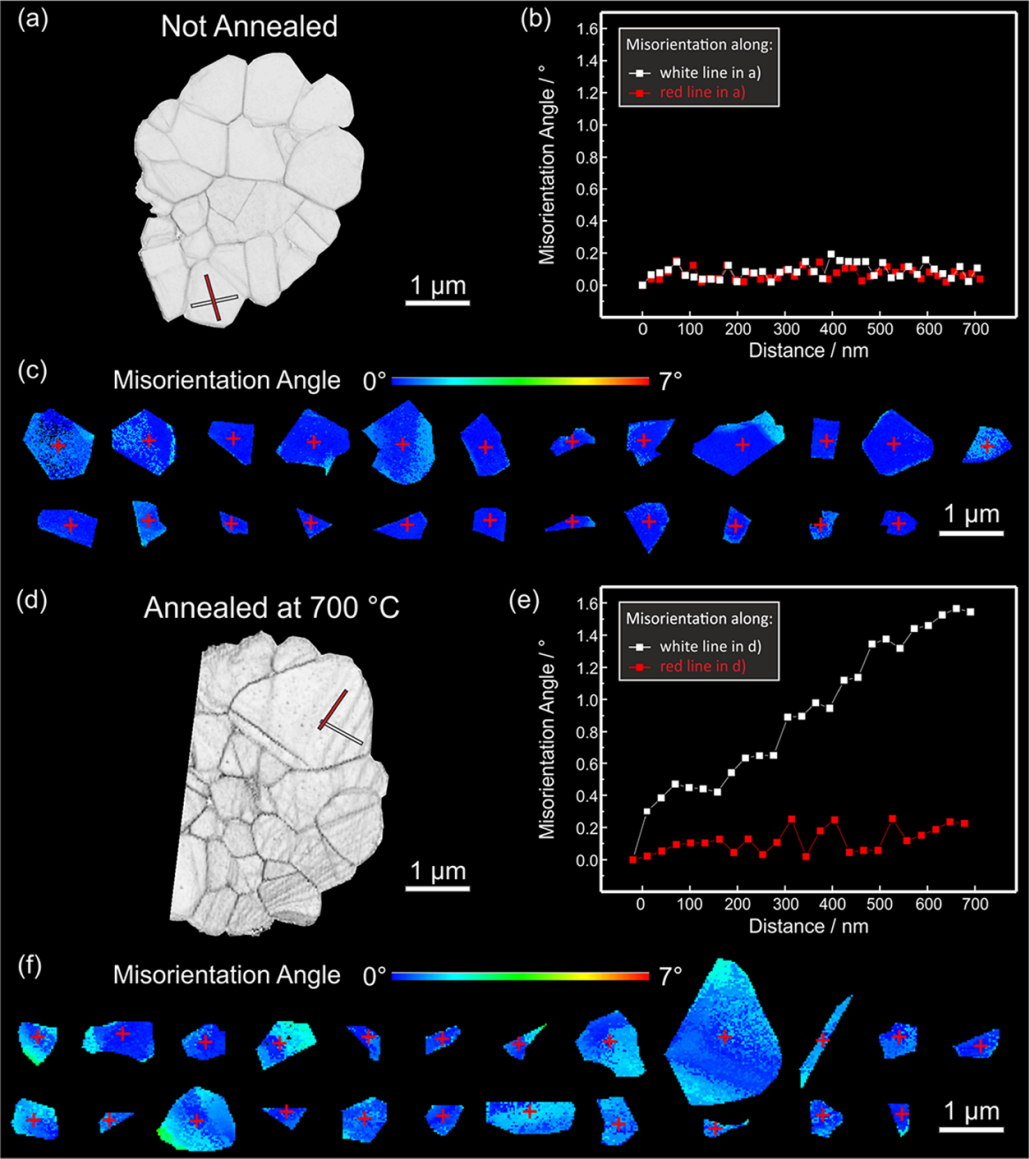
(a) SPED CCM of a u-LNO particle. (b) Graph depicting the misorientation of adjoining diffraction patterns in the SPED data set along the white and red lines drawn in (a). (c) Individual primary particles of the LNO particle depicted in (a) are color-coded to visualize the misorientation angle relative to the center of the grain. (d) SPED CCM of an LNO-700 particle. (e) Graph depicting the misorientation of adjoining diffraction patterns in the SPED data set along the white and red lines drawn in (d). (f) Individual primary particles of the LNO particle depicted in (d) are color-coded to visualize the misorientation angle relative to the center of the grain.

Two possible explanations come to mind for the presence of low-angle misorientations in annealed LNO, and why they are more pronounced in LNO-700 compared to LNO-600. The first relates to lithium reinsertion during annealing. In addition to lithium re-entering the lattice, Ni^2+^ ions migrate from lithium 3a sites back to 3b sites. This process is expected to be more pronounced at 700 °C than at 600 °C, as also supported by our findings of higher lithium occupancy in LNO-700. Such structural rearrangements may introduce internal stress. A second explanation involves contraction during cooling, which can induce thermal residual stress that manifests as low-angle misorientations. Since the LNO samples were not cooled in a controlled manner but simply left to cool with the oven off, the cooling ramp was likely faster for LNO-700 than for LNO-600, possibly resulting in stronger thermal stresses. While we tend to favor the latter explanation, we acknowledge that both effects may contribute, and a definitive conclusion cannot be drawn from the present data set. Internal stress in CAMs is generally considered undesirable, as it may lead to mechanical failure and reduce the capacity of the battery.^82,83^ Interestingly, in our study, the presence of low-angle misorientations does not correlate with reduced initial performance—in fact, LNO-700 shows the highest discharge capacity among the samples, despite exhibiting the strongest internal misorientation. While this suggests no immediate detrimental effect in early cycling, our study does not provide insight into long-term cycling behavior, which could still be affected by such structural distortions. To investigate this, a dedicated study examining the origin of these misorientations—e.g., by using a controlled cooling ramp after annealing—and their influence on the long-term cycling capabilities of the cells is needed. For this purpose, full-cell tests are required, which go beyond the scope of this present study. We highly recommend further analysis of this topic, as a two-step temperature-swing synthesis otherwise shows promising potential in improving CAMs.

## 4 Conclusions

In this study, we investigated the impact of a two-step temperature-swing synthesis on the electrochemical performance and structure of LiNiO_2_ particles with large primary grains, compared to LNO particles (u-LNO) that did not undergo this process. We selected two annealing temperatures, 600 °C (LNO-600) and 700 °C (LNO-700), for our investigation.

Our findings show that the additional annealing step at elevated temperatures smooths the particle surfaces, reduces the amount of reactive lithium on the surface, and simultaneously increases the lithium occupancy within the crystal lattice. These changes result in a higher discharge capacity for the annealed samples during the initial cycles, with the effects being slightly more pronounced in LNO-700 compared to LNO-600.

Structural investigations using several complementary transmission electron microscopy techniques (STEM, EELS, SPED) revealed that a rock-salt layer forms on the surface of the LNO particles at higher annealing temperatures. Although the annealing temperature does not seem to affect the number of planar defects in the samples, LNO-700 exhibits a high degree of low-angle misorientations within its primary grains, which is not observed in u-LNO and to that extent in LNO-600. This low-angle misorientation likely indicates internal stress caused by thermal contraction during cooling.

Despite the presence of a rock-salt layer on the surface and internal stress, which are generally considered detrimental to CAM performance, LNO-700 demonstrates the highest initial discharge capacity. This suggests that the formation of a rock-salt layer prior to cycling may not be as detrimental to the battery performance as widely assumed. Additionally, the benefits of a smoother surface, reduced amounts of reactive lithium on the surface, and a higher lithium occupancy might outweigh the negative effects associated with the rock-salt layer and internal stress during the first three cycles. Altogether, our findings underscore a clear advantage of a two-step temperature-swing synthesis over unannealed LNO. Whether the positive benefits continue to outweigh the adverse effects throughout the battery’s life cycle will need to be evaluated in long-term cycling experiments.

Additionally, our study highlights the importance of a comprehensive TEM-based approach, integrating multiple complementary techniques alongside electrochemical data, to evaluate the structural and morphological evolution of CAM particles during different sintering procedures.

## Supplementary Material



## Notes

PXRD diffractograms of u-LNO, LNO-600, and LNO-700; screenshot of the indexing software, depicting the best match of the input phases at different scan positions; HAADF STEM images marking the regions of the EELS scans shown in Figure 4; additional SPED correlation coefficient maps of u- LNO LNO-700 particles for better statistics; SPED correlation coefficient map of LNO-600; EELS peak intensities and calculated Li K-edge to Ni M-edge as well as Li surface-to-bulk ratios; and additional Information on PXRD parameters and Rietveld refinement (PDF)

## Abbreviations

CAM: cathode active material
LNO: LiNiO_2_
TEM: transmission electron microscope
STEM: scanning transmission microscope
SEM: scanning electron microscopy
SPED: scanning precession electron diffraction
EELS: electron energy loss spectroscopy
FIB: focused ion beam
PXRD: powder X-ray diffraction
HAADF: high-angle annular darkfield
CCM: correlation coefficient map
SE: secondary electrons
TB: Twin boundary
SSB: solid-state battery
TLD: through-the-lense detector
