# Supplementary material for: Effect of a Two-Step Temperature-Swing Synthesis on Coarse-Grained LiNiO2 Secondary Particles Characterized by Scanning Transmission Electron Microscopy
Source: Chem Mater. 2025 May 16;37(11):3993–4004. doi: 10.1021/acs.chemmater.5c00108 (PMC12160585; doi:10.1021/acs.chemmater.5c00108)
Supplement: Supplementary file 1 [file cm5c00108_si_001.pdf]

Supplementary Information

Effect of a Two-Step Temperature-Swing Synthesis on Coarse-Grained $LiNiO_2$ Secondary Particles Characterized by Scanning Transmission Electron Microscopy

Thomas Demuth[1], Philipp Kurzhals[2,3], Shamail Ahmed[1], Felix Riewald[3], Michael Malaki[1], Johannes Haust[1], Andreas Beyer[1], Jürgen Janek[2], Kerstin Volz[1,]*

[1]Materials Science Center (WZMW) and Department of Physics, Philipps University Marburg, 35032 Marburg, Germany

[2]Institue of Physical Chemistry and Center for Materials Research, Justus-Liebig-University (JLU), 35392 Giessen, Germany

[3]BASF SE, New Battery Materials and Systems, 67506 Ludwigshafen am Rhein, Germany

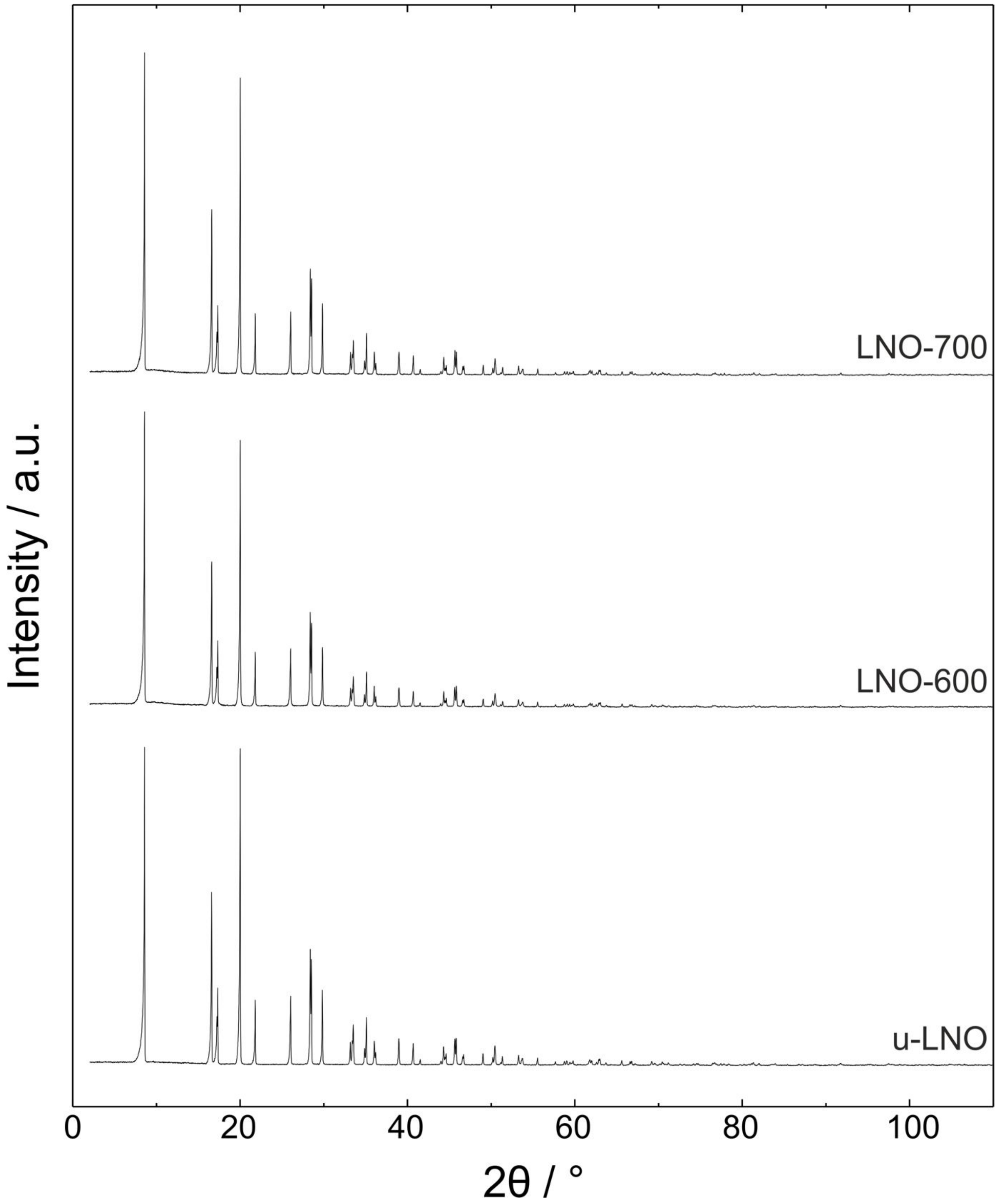

Figure S1: PXRD diffractograms of u-LNO, LNO-600, and LNO-700.

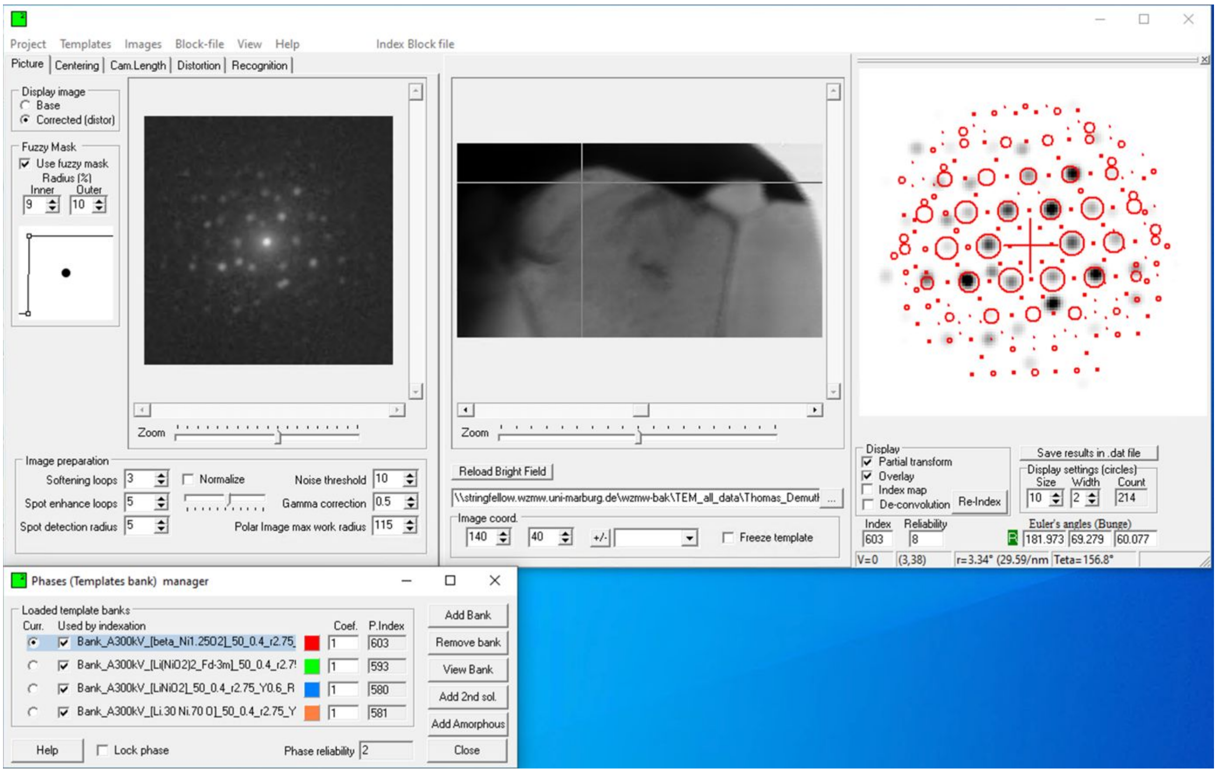
Project Templates Images Block-file View Help Index Block file
Picture Centering Cam.Length Distortion Recognition
Display image
Base
Corrected (distor)
Fuzzy Mask
Use fuzzy mask
Radius (%)
Inner Outer
9 10
Zoom
Image preparation
Softening loops 3
Normalize
Noise threshold 10
Spot enhance loops 5
Gamma correction 0.5
Spot detection radius 5
Polar Image max work radius 115
Reload Bright Field
\\stringfellow.wzmw.uni-marburg.de\wzmw-bak\TEM_all_data\Thomas_Demutl
Image coord.
140 40 +/-
Freeze template
Display
Partial transform
Overlay
Index map
De-convolution
Re-Index
Save results in .dat file
Display settings (circles)
Size Width Count
10 2 214
Index Reliability
603 8
Euler's angles (Bunge)
181.973 69.279 60.077
V=0 (3,38) r=3.34° (29.59/nm Teta=156.8°
Phases (Templates bank) manager
Loaded template banks
Curr. Used by indexation Coef. P.Index
Bank_A300kV_[beta_Ni1.25O2]_50_0.4_r2.75_ 1 603
Bank_A300kV_[Li(NiO2)2_Fd-3m]_50_0.4_r2.7! 1 593
Bank_A300kV_[LiNiO2]_50_0.4_r2.75_Y0.6_R 1 580
Bank_A300kV_[Li.30 Ni.70 O]_50_0.4_r2.75_Y 1 581
Add Bank
Remove bank
View Bank
Add 2nd sol.
Add Amorphous
Help
Lock phase
Phase reliability 2
Close

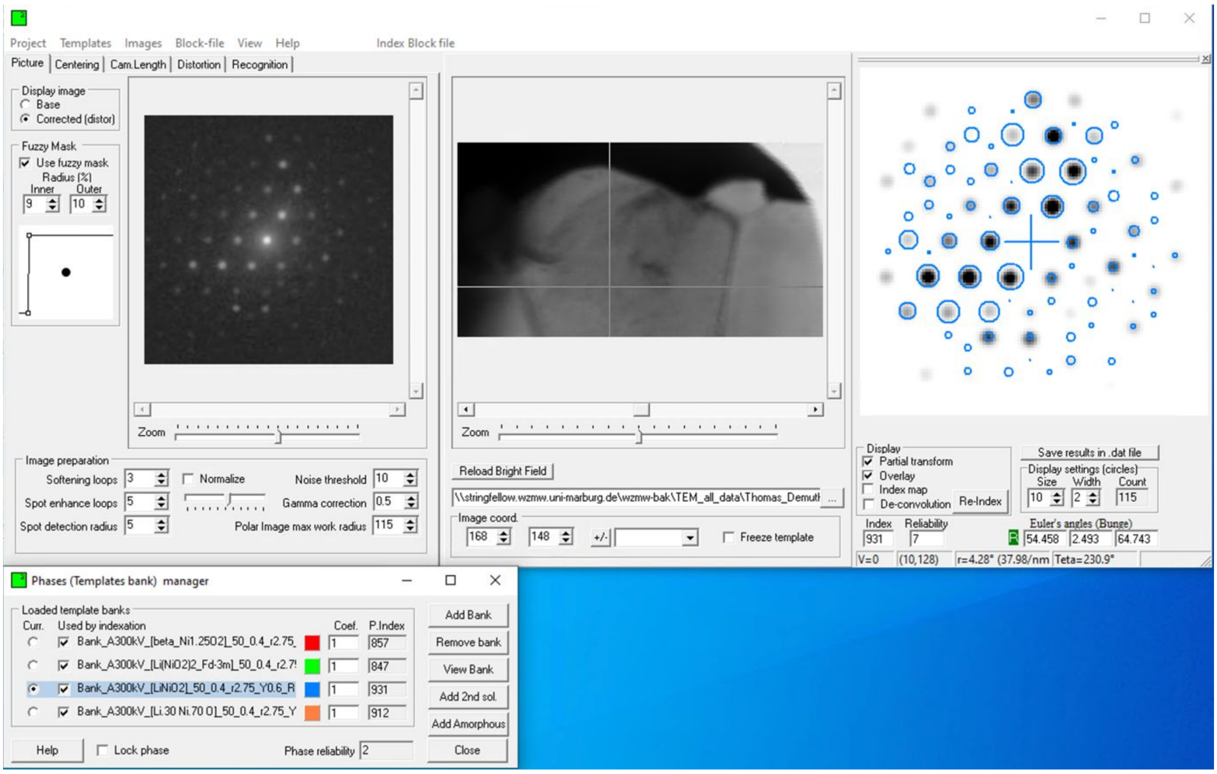
Project Templates Images Block-file View Help Index Block file
Picture Centering Cam.Length Distortion Recognition
Display image
Base
Corrected (distor)
Fuzzy Mask
Use fuzzy mask
Radius (%)
Inner Outer
9 10
Zoom
Image preparation
Softening loops 3
Normalize
Noise threshold 10
Spot enhance loops 5
Gamma correction 0.5
Spot detection radius 5
Polar Image max work radius 115
Reload Bright Field
\\stringfellow.wzmw.uni-marburg.de\wzmw-bak\TEM_all_data\Thomas_Demutl
Image coord.
168 148 +/-
Freeze template
Display
Partial transform
Overlay
Index map
De-convolution
Re-Index
Save results in .dat file
Display settings (circles)
Size Width Count
10 2 115
Index Reliability
931 7
Euler's angles (Bunge)
54.458 2.493 64.743
V=0 (10,128) r=4.28° (37.98/nm Teta=230.9°
Phases (Templates bank) manager
Loaded template banks
Curr. Used by indexation Coef. P.Index
Bank_A300kV_[beta_Ni1.25O2]_50_0.4_r2.75_ 1 857
Bank_A300kV_[Li(NiO2)2_Fd-3m]_50_0.4_r2.7! 1 847
Bank_A300kV_[LiNiO2]_50_0.4_r2.75_Y0.6_R 1 931
Bank_A300kV_[Li.30 Ni.70 O]_50_0.4_r2.75_Y 1 912
Add Bank
Remove bank
View Bank
Add 2nd sol.
Add Amorphous
Help
Lock phase
Phase reliability 2
Close

Figure S2: Screenshot of the Indexing Software depicting the best match of the input phases to the recorded diffraction pattern at the positions of the cross. For clarity only the most prominent phases in Figure 4 ($LiNiO_2$, $Li(NiO_2)_2$, β-$Ni_{0.25}NiO_2$, $Li_{0.3}Ni_{0.7}O$) were chosen as input. It is apparent, that while the β-$Ni_{0.25}NiO_2$ phase (red) is the best match of the input phases, it is not a perfect match, as many diffraction spots are not matched. This is different for the layered $LiNiO_2$ phase (blue), which fits nearly perfectly to the recorded pattern.

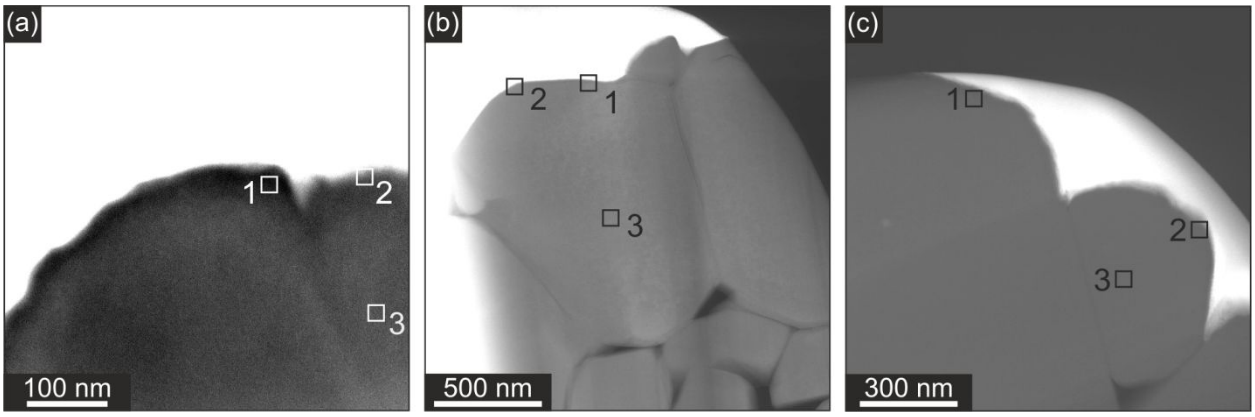

Figure S3: HAADF STEM images marking the regions of the EELS scans shown in Figure 4. (a) u-LNO, (b) LNO-600, (c) LNO-700.

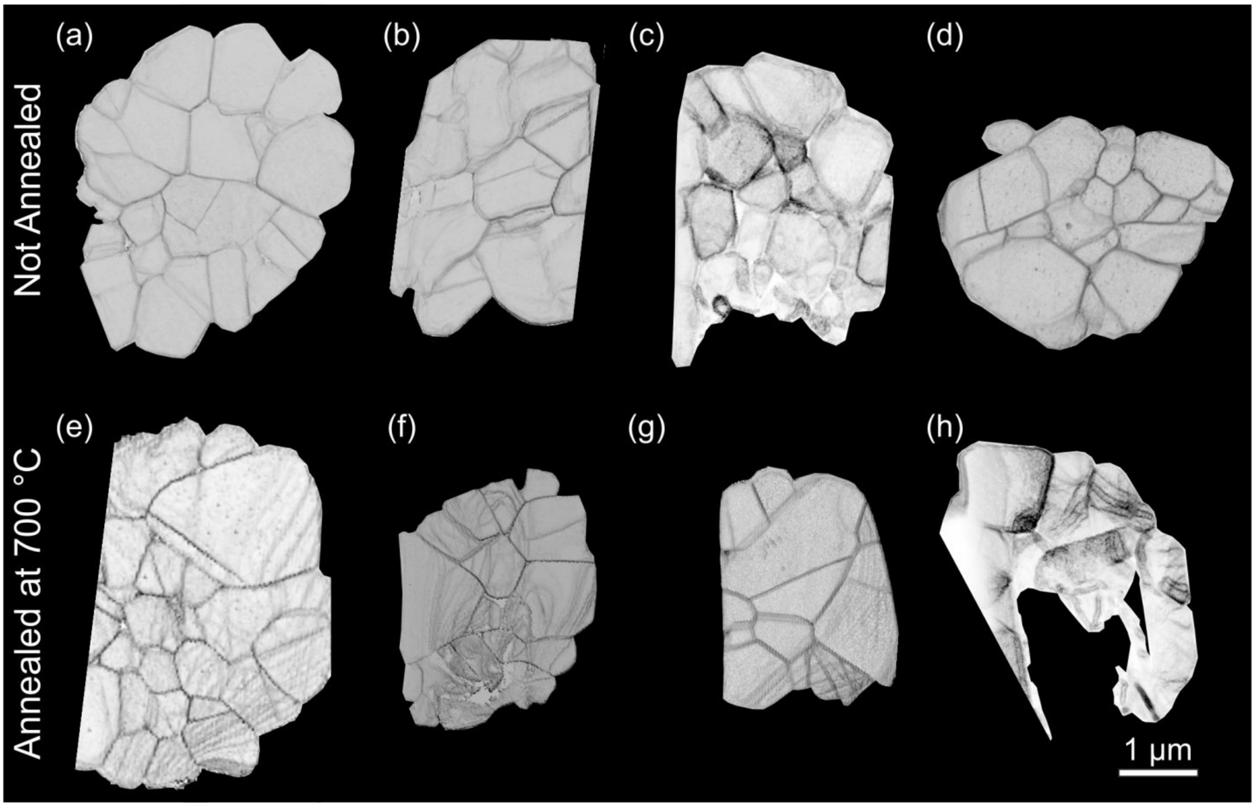

Figure S4: SPED correlation coefficient maps (CCM) of (a) – (d) u- LNO and (e) – (h) LNO-700 particles.

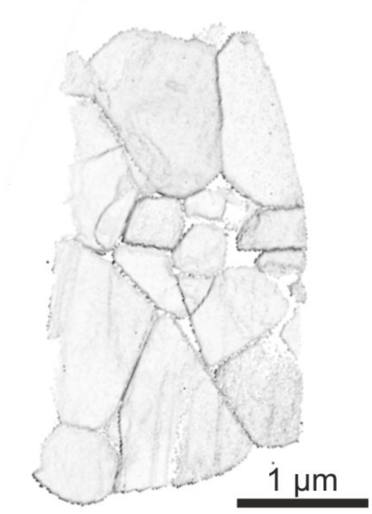

Figure S5: SPED CCM of an LNO-600 particle.

Table S1: EELS peak intensities and calculated Li K-edge to Ni M-edge as well as Li surface-to-bulk ratios. The vastly different amounts of counts originate from the data acquisition. For u-LNO and LNO-700, the EELS spectra included the zero-loss peak, which leads to an overall reduced intensity of the low loss spectrum to oversaturate the camera compared to LNO-600, where the zero-loss peak was recorded separately from the core-loss data.

**Sample**	**Region No.**	**Region Type**	**Li K-edge Counts**	**Ni M_3-edge Counts**	**Li K-edge/ Ni M-edge**	**Li surface / Li bulk**
u-LNO	1	bulk	34 ± 1	73 ± 1	0.47	0.51
	2	surface	57 ± 1	73 ± 1	0.78	0.84
	3	surface	68 ± 1	73 ± 1	0.93	-
LNO-600	1	bulk	689 ± 10	2523 ± 10	0.27	0.38
	2	surface	1049 ± 10	2523 ± 10	0.42	0.59
	3	surface	1790 ± 10	2523 ± 10	0.71	-
LNO-700	1	bulk	15 ± 1	64 ± 1	0.23	0.31
	2	surface	35 ± 1	64 ± 1	0.55	0.73
	1	surface	48 ± 1	64 ± 1	0.75	1.00

Supplementary Material 1: Additional Information on PXRD Parameters and Rietveld Refinement

The NIST 660c LaB6 is used to calibrate and determined the instrumental parameters of XRD.
The Li/Ni disorder was calculated from Rietveld refinements (the refined occupancy of Ni in the Li 3b / amount of 3a sites * 100%)

Rietveld Refinement of NIST 660c

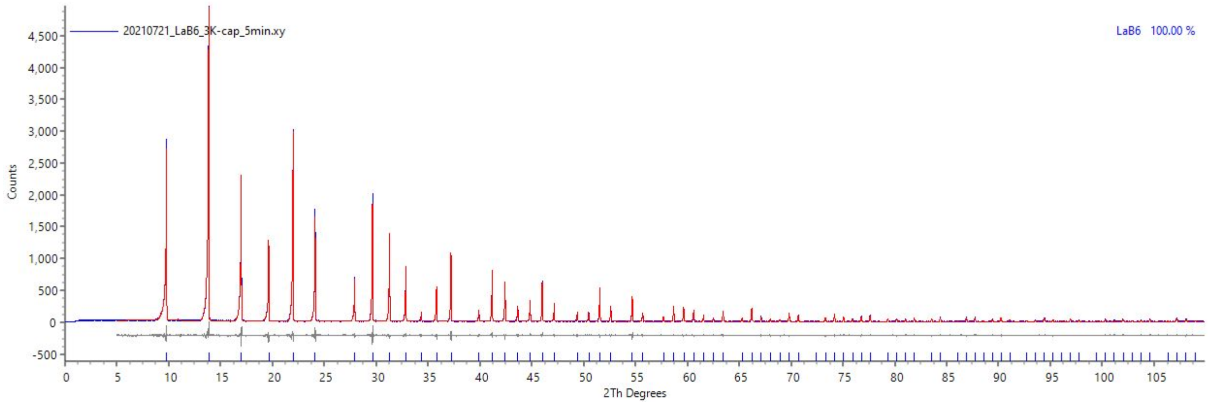

u-LNO

Atom	Site	x	y	z	Occ.
**Li**	3a	0.000	0.000	0.000	0.918
**Ni**	3a	0.000	0.000	0.000	0.082
**Ni**	3b	0.000	0.000	0.500	1.00
**O**	6c	0.000	0.000	0.244	1.00

a (Å)	2.8785±0.0002
c (Å)	14.1955±0.0009
Volume (Å^3)	101.86±0.01
Crystallite Size (nm)	67.30273±8.63282
Li Occupancy	0.918±0.004

LNO-600

Atom	Site	x	y	z	Occ.
**Li**	3a	0.000	0.000	0.000	0.98374
**Ni**	3a	0.000	0.000	0.000	0.01626
**Ni**	3b	0.000	0.000	0.500	1.00
**O**	6c	0.000	0.000	0.244	1.00

a (Å)	2.878657±0.000189
c (Å)	14.213630±0.000897
Volume (Å^3)	102.004±0.015
Crystal Density (g/cm^3)	4.81229±0.01122
Crystallite Size (nm)	285.37725±25.8984
Strain (Δd/d)	0.00027±0.0000
Rwp (%)	8.78
Li Occupancy	0.98374±0.00363

LNO-700

Atom	Site	x	y	z	Occ.
**Li**	3a	0.000	0.000	0.000	0.99
**Ni**	3a	0.000	0.000	0.000	0.01
**Ni**	3b	0.000	0.000	0.500	1.00
**O**	6c	0.000	0.000	0.244	1.00

a (Å)	2.878380±0.000141
c (Å)	14.203231±0.000681
Volume ($Å^3$)	101.909±0.012
Crystal Density (g/cm^3)	4.79812±0.01041
Crystallite Size (nm)	610.92041±94.125
Strain (Δd/d)	0.00026±0.0000
Rwp (%)	8.14
Li Occupancy	0.99000±0.00390
